# Exploring the significance of caspase-cleaved tau in tauopathies and as a complementary pathology to phospho-tau in Alzheimer’s disease: implications for biomarker development and therapeutic targeting

**DOI:** 10.1186/s40478-024-01744-9

**Published:** 2024-02-28

**Authors:** Liara Rizzi, Lea T. Grinberg

**Affiliations:** 1 Memory and Aging Center, Department of Neurology, Sandler Neurosciences Center, University of California San Francisco, 675 Nelson Rising Lane, San Francisco, CA 94158 USA; 2 Department of Neurology, University of Campinas (UNICAMP), Campinas, SP Brazil; 3 Department of Pathology, LIM-22, University of São Paulo Medical School, São Paulo, SP Brazil; 4 Department of Pathology, University of California San Francisco, San Francisco, CA USA

**Keywords:** Caspases, Tau, Tauopathies, Alzheimer’s disease, Diagnosis, Postmortem, Biomarkers

## Abstract

Tauopathies are neurodegenerative diseases that typically require postmortem examination for a definitive diagnosis. Detecting neurotoxic tau fragments in cerebrospinal fluid (CSF) and serum provides an opportunity for in vivo diagnosis and disease monitoring. Current assays primarily focus on total tau or phospho-tau, overlooking other post-translational modifications (PTMs). Caspase-cleaved tau is a significant component of AD neuropathological lesions, and experimental studies confirm the high neurotoxicity of these tau species. Recent evidence indicates that certain caspase-cleaved tau species, such as D13 and D402, are abundant in AD brain neurons and only show a modest degree of co-occurrence with phospho-tau, meaning caspase-truncated tau pathology is partially distinct and complementary to phospho-tau pathology. Furthermore, these caspase-cleaved tau species are nearly absent in 4-repeat tauopathies. In this review, we will discuss the significance of caspase-cleaved tau in the development of tauopathies, specifically emphasizing its role in AD. In addition, we will explore the potential of caspase-cleaved tau as a biomarker and the advantages for drug development targeting caspase-6. Developing specific and sensitive assays for caspase-cleaved tau in biofluids holds promise for improving the diagnosis and monitoring of tauopathies, providing valuable insights into disease progression and treatment efficacy.

## Introduction

Tauopathies are associated with high morbidity and mortality, and lack of effective treatment options [1]. They are classically diagnosed based on the detection of phospho-tau inclusions in the brain, which requires a biopsy or autopsy [2]. In recent years, efforts have been made to create fluid biomarkers based on phospho-tau to non-invasively detect pathology in living patients. These biomarkers have proven useful in diagnostic screening, particularly in the context of Alzheimer’s disease (AD) [3]. Nevertheless, tauopathies exhibit pathological tau inclusions with post-translational modifications (PTMs) beyond phosphorylation. Caspase-cleaved tau is highly neurotoxic [4], and it is present in AD and other tauopathies. Tau protein can undergo cleavage by various caspases, including caspases 1, 2, 3, 6, 7, and 8. Among the caspase-cleaved tau species studied in the context of neurodegenerative diseases, tau D421 is the most investigated. Tau D421 can result from cleavage by multiple caspases. Limited studies on cerebrospinal fluid (CSF) involving caspase-cleaved tau indicate that these fragments are released and can be identified in the CSF, potentially exhibiting a meaningful correlation with clinical deterioration [5, 6]. Recent findings indicate that caspase-6 cleaved tau D13 and D402, commonly present in AD neurons, but rarely in 4-repeat tauopathies, only partially overlap with phospho-tau in the same neuron [7]. This implies that caspase-cleaved tau can signal tau pathology distinct from phospho-tau, highlighting a pathway that cannot be identified solely through phospho-tau-based biomarkers. Despite substantial evidence pointing to the relevance of caspase-cleaved tau in the pathogenesis of certain tauopathies, this disease pathway continues to be inadequately explored. This review will examine the evidence of caspase-cleaved tau contribution to tauopathies, with a specific emphasis on the more recent advancements related to caspase-6 cleaved tau. Furthermore, we will delve into the potential for the development of biomarkers and drugs based on these research findings.

## Tauopathies

Tauopathy is an umbrella term encompassing more than 20 well-defined, progressive neurodegenerative entities characterized by abnormal accumulation of protein tau in neurons and glial cells [8]. Sporadic tauopathies are classified based on the pattern of morphological distribution of the inclusions, what types of cells accumulate tau, and the biochemical composition of tau inclusions, namely predominance of three microtubule-binding repeats (3R) tau, four microtubule-binding repeats (4R) tau or a combination of both (3R/4R) [8]. Examples of 3R/4R tauopathies include AD and chronic traumatic encephalopathy (CTE). Pick’s disease (PiD) falls into the 3R category. Progressive supranuclear palsy (PSP), corticobasal degeneration (CBD), globular glial tauopathy (GGT), argyrophilic grain disease (AGD), and aging-related tau astrogliopathy (ARTAG) belong to the 4R category. Familial tauopathies exhibit distinct clinicopathological phenotypes depending on the specific microtubule-associated protein tau (MAPT) mutation [9].

The frequencies of tauopathies vary among the populations and diagnostic criteria applied. AD is the most common neurodegenerative condition with the accumulation of abnormal tau in the brain [10]. AGD and ARTAG are prevalent, affecting up to 50% of individuals who come to autopsy at age 80 and older. However, AGD and ARTAG are considered tau accumulation with minimal clinical correlates [11, 12]. Progressive supranuclear palsy and corticobasal degeneration, the most prevalent pathogenic sporadic 4R-tauopathies, have a pooled prevalence rate of 7.1 and 2.3 per 100,000 individuals, respectively [13], but a recent clinicopathological study suggests that PSP prevalence is much higher [14].

Tau protein isoforms can undergo various post-translational modifications (PTM), such as acetylation, ubiquitination, phosphorylation, glycation, glycosylation, SUMOylation, methylation, oxidation, truncation, and nitration [15], resulting in species with different consequences for tau assembly, function, and accumulation [16]. Some tau PTMs produce neurotoxic fragments of various lengths and divergent pathological effects [17]. Phospho-tau species represent a ubiquitous post-translational modification (PTM) present in all tauopathies. Consequently, the detection of phospho-tau inclusions is the preferred method for the neuropathological diagnosis of tauopathies [8]. While other tau PTMs may be identified in various tauopathies, these inclusions are generally considered to be present only in a subgroup of cells that already exhibit phospho-tau inclusions.

Tau truncation by proteases - such as caspases, the theme of this review - leads to significant alterations in its structure and function, resulting in the loss- or gain of function depending on the truncation site [18]. Some protease-mediated tau truncations have a critical role in molecular events leading to pathological changes in tauopathies [19].

## Caspases

Caspases, a large group of cysteine proteases commonly associated with inflammation and apoptosis [20], participate in tau proteolysis. Caspases cleave substrates at specific aspartic acid (Asp) residues [21, 22]. In their inactive proenzyme form, they reside in the cytosol and are activated by dimerization or proteolytic cleavage [23]. Once activated, they proteolytically degrade proteins by altering their cellular structure and functions [23]. The caspase family includes at least 14 enzymes [22]. Classically, caspases are divided into upstream initiators and downstream effectors. Upstream caspases 1, 4, 5, 11, 12, and 13 trigger inflammatory processes by cytokine activation, while 2, 8, 9, and 10 are associated with apoptosis initiation. Upon activation, upstream caspases initiate an amplification cascade that activates downstream effector caspases. Downstream caspases 3, 6, and 7 are effectors of apoptosis, while 14 is involved in cytokine maturation [24].

## Caspase-cleaved tau in Alzheimer’s disease

**Fig. 1:**
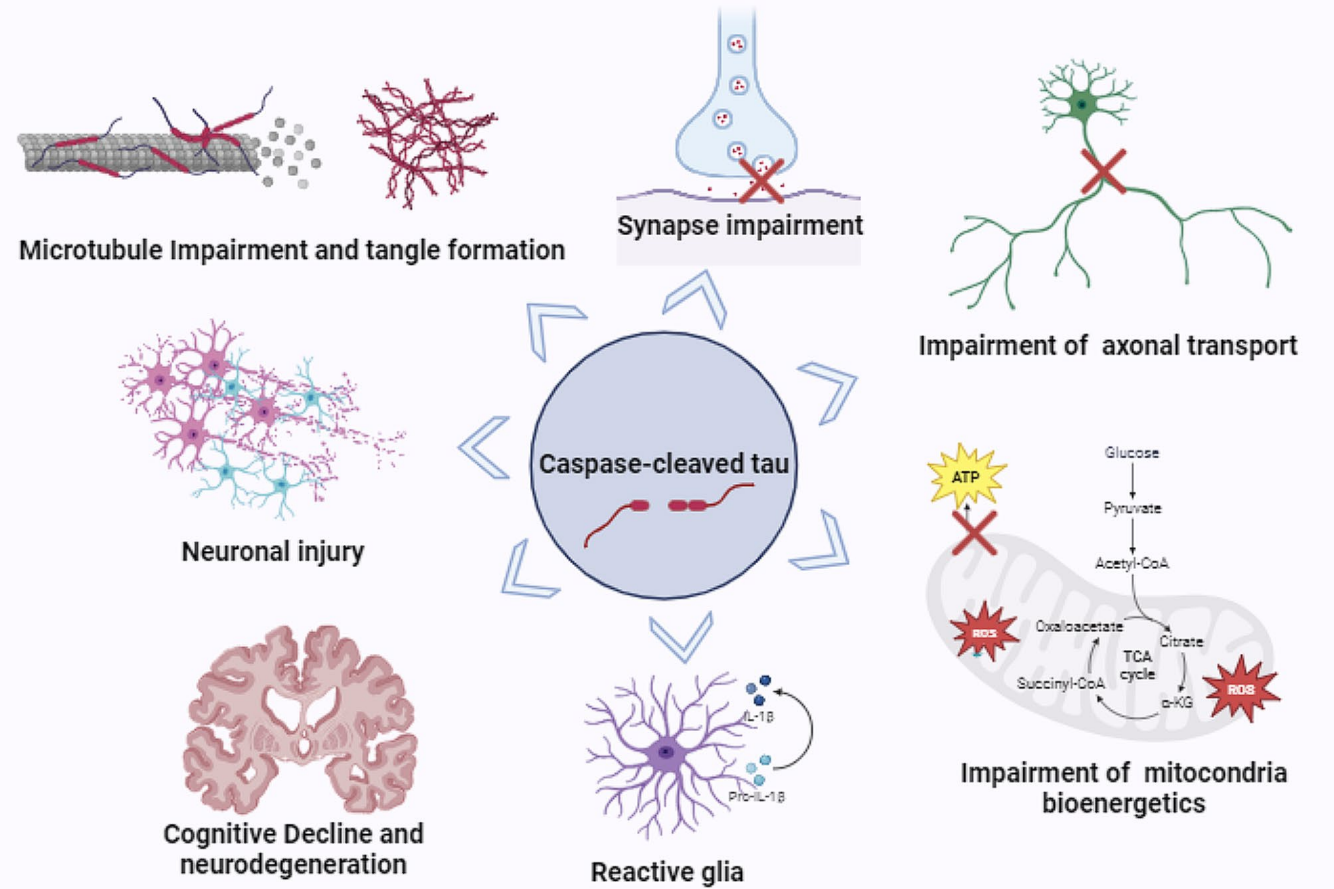
Pathological Mechanisms induced by Caspase-Cleaved Tau

Studies on caspase-cleaved tau have mainly focused on AD and show that tau can be cleaved at multiple sites by caspases resulting in carboxy or amino truncations [24–26] (Table 1). Cleavage of tau’s C-terminus or N-terminus by caspases leads to impairments in mitochondrial bioenergetics, weakening of axonal transport, neuronal injury, and cognitive decline [25]. Besides, these truncations contribute to the formation of amyloid-β plaques and intracellular neurofibrillary tangles [27] (Fig. 1).

**Table 1:** Sites where caspases cleave tau protein

Caspase	Tau Cleavage Site(s)	Reference
Caspase-1	D421	Gamblin et al. [28]
Caspase-2	D65 D314	Reinhardt et al. [29] Zhao et al. [30]
Caspase-3	D25 D421	Corsetti et al. [31] Gamblin et al. [28]
Caspase-6	D13 D402 D421	Horowitz et al. [32] Guo et al. [23] Gamblin et al. [28] Theofilas et al. [7]
Caspase-7	D421	Gamblin et al. [28]
Caspase-8	D421	Gamblin et al. [28]

### Caspase-3

**Fig. 2:**
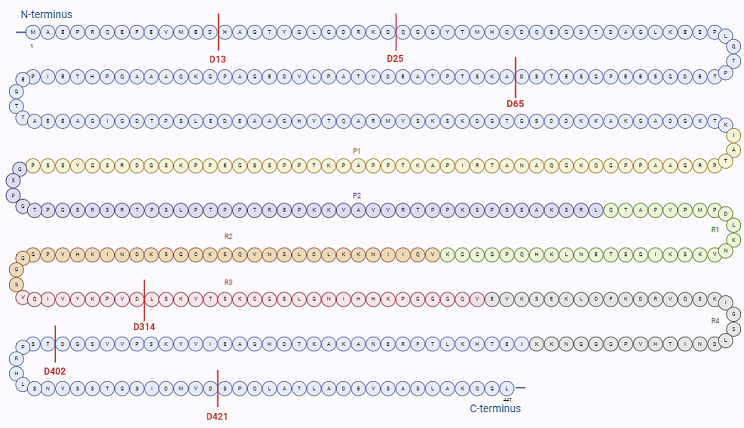
Putative sites caspase-cleaved tau. Caspases 1, 3, 6, 7, and 8 cleave tau at D421. Caspase-2 cleaves tau also at D65 and D314, caspase-3 cleaves tau also at D25, caspase-6 cleaves tau also at D402 and D13. Tau consists of four domains: the projection domain (M1–Y197), a proline-rich region (P1 and P2), the microtubule-binding repeats (R1, R2, R3, R4), and a C-terminus domain (K369–L441). Amino acids 1-441

Caspase-3 appears to cleave tau protein after Asp25 or Asp421 [31]. Caspase-cleaved tau in its C-terminal tail at Asp421, which removes 20 amino acids from tau C-terminal, also known as TauC3, or tau D421, were the focus of the first studies on tauopathies [28, 33]. I*n vitro*, caspase-3-cleaved tau at Asp421 assembles into filaments more rapidly than wild-type tau [28]. TauC3 may contribute to the propagation of tau pathology by inducing mitochondrial fragmentation and bioenergetics dysfunction in neuronal cells [25, 34, 35], neurite loss in neuronal cultures, and increasing tau polymerization and aggregation in vitro [28, 36]. In vivo, multiphoton imaging in a living tau transgenic mice model (Tg4510 strain), de Calignon et al. detected tau D421, generated by caspase-3-cleavage preceding neurofibrillary tangle pathology and determined that tau D421 promoted the formation of neurofibrillary tangles [37, 38]. Others have also shown that tau cleavage precedes tau tangle pathology [26] and tau oligomer formation in transgenic mice expressing human TauC3 [39]. Moreover, in tau knockout mice, the proportion of caspase-3-cleaved tau at Asp421 doubled in the hippocampus during aging. In this case, cleaved tau induced a toxic gain of function that delayed axonal transport and led to region-specific dendritic atrophy in CA1 neurons [33]. This level of neurotoxicity was confirmed in studies with non-transgenic (male C57BL/6J) mice, caspase-3-cleaved tau increases in the forebrain in an aged-related manner and correlates to cognitive deficits [40]. Likewise, caspase-3-cleaved tau is associated with neurofibrillary tangles and cognitive decline in human brains [26]. Notably, it became clear that caspases 1, 6, 7, and 8 also cleave tau at Asp421 [28](Fig. 2).

In humans, tau D421 is found in AD and other tauopathies. Interestingly, E3 ubiquitin ligase CHIP binds the latent C-terminal at tau Asp421. Loss of CHIP expression in AD coincides with the accumulation of tau Asp421, suggesting an interaction between caspases and protein homeostasis in AD and other tauopathies and a therapeutic opportunity [41].

### Caspase-6

Evidence that caspase-6 activation is associated with a protracted type of cell death in serum-deprived human primary neurons [42] and renders neurons susceptible to oxidative stress, resulting in either immediate or delayed apoptosis [43], as opposed to the other effector caspases that instead lead to rapid induction of apoptosis. These unique characteristics of caspase-6, less studied than caspase-3, have drawn attention to its potential importance. Caspase-6 in inactivated form is ubiquitous in the human fetal brain and peripheral tissues showing its importance for fetal development [44]. In normal conditions, the human adult brain expresses low levels of caspase 6. However, neuronal activation of caspase-6 is an early event in AD and correlates with adverse clinical outcomes. Increased caspase-6 activity in the anterior olfactory nucleus reflected the degeneration in the entorhinal cortex (affected in Braak stage 1) and correlated with tau pathology in human AD olfactory bulb brain Sect. [45]. Also, in aged non-cognitively impaired individuals, the level of caspase-6 in the entorhinal cortex and CA1 negatively correlates with cognitive domains initially affected in AD [46]. In a study probing the locus coeruleus and dorsal raphe nucleus, brain regions among the first to develop AD-tau pathology, levels of caspase-6 activation in neurons associated with increased Braak staging and burden of neurofibrillary tangles positive for phospho-tau [47]. Levels of caspase-6-cleaved tau are inversely correlated with global cognitive scores in non-demented individuals, supporting tau that cleavage by active caspase-6 may be an early event in AD pathophysiology [21, 48, 49]. In addition to Asp421, caspase-6 cleaves tau at other sites, including Asp402 and Asp13 [23, 32] (Fig. 2). Caspase-6 is particularly efficient in cleaving tau at Asp13 [50]. Caspase-6-cleavage of tau at Asp13 generates two fragments [32]: 1–13 with unknown function and 14–441 with a role in tangle maturation [51, 52]. Caspase-6- cleavage of tau at Asp402 generates a 1-402 fragment, associated with neurodegeneration, and the 403–441 with unknown functions [52]. Altogether, both active caspase-6 and tau truncated at Asp402 and Asp13 are present in neurofibrillary tangles, neuritic plaques, and neuropil threads in sporadic and familial AD but absent in brains without AD pathology [7, 23, 48, 53].

Despite confluent evidence of the role of caspase-6 activation and caspase-6-cleaved in AD pathogenesis [48, 54], only recently monoclonal antibodies against these tau specimens became available to directly investigate the frequency of tau D13 and D402 in tauopathies. Theofilas et al. [7] probed postmortem human brain tissue with a recently developed 5-plex immunohistochemistry with monoclonal novel neoepitope monoclonal antibody against caspase-6 cleaved tau (D402 and D13). The use of multiplex immunostaining allowed these researchers to detect that the number of neurons positive for caspase-6 cleaved tau and phospho-tau in AD is equivalent. However, the overlapping is only 45%. It suggests that currently used antibodies do not flag a significant portion of neurons with tau pathology to label pathological tau in postmortem studies and fluid-based biomarkers based on phospho-tau.

### Caspase-1

Caspase-1 has been reported to cleave tau at Asp421 [28], but caspase-1 impact in AD is more likely related to abnormal activation of the NLR family pyrin domain containing 1 (Nlrp1) inflammasome in AD neurons [55]. The Nlrp1 inflammasome is sequentially activated, leading to the activation of caspase-1. Subsequently, caspase-1 triggers caspase-6-mediated neurodegeneration and IL-1β-mediated glial response [56].

Intense active expression of caspase-1 has been detected in the brains of individuals with mild cognitive impairment and dementia due to AD [57]. A selective inhibitor of caspase-1, VX-765, significantly rescued spatial learning, and memory impairments and reduced tau hyperphosphorylation in the brains of senescence-accelerated mouse prone 8 (SAMP8) mice [58]. While the precise role of caspase-1 in AD neuropathology remains unclear, compelling evidence suggests that targeting caspase-1 could be a viable therapeutic approach for addressing AD and possibly other tauopathies [58], alone or combination with drugs targeting Nlrp1 and caspase-6 [59].

### Caspase-2

Caspase-2 has been reported to cleave tau at D314 and D65 [29, 30] (Fig. 2). Caspase-2-cleaved tau reversibly impairs memory function in animal and cellular models of tauopathies due to accumulation in dendritic spines and attenuation of synaptic transmission [60]. However, others have shown that caspase-2-cleaved tau shows increased aggregation and accumulation only in vitro, despite strong RNA expression in vivo models, suggesting efficient clearance. In vivo and in vitro, caspase-2-cleaved tau is also recognized by the ubiquitin E3 ligase CHIP, contributing to faster degradation of caspase-2-generated tau fragments [29]. Levels of truncated tau at D314 are elevated in the inferior temporal gyrus of AD and MCI individuals [61]. Incidentally, caspase-2-cleaved tau fragments have been found in other diseases such as Lewy body disease [62], and Huntington’s disease [63], showing that they are not specific to AD or tauopathies [64].

## Caspase-cleaved tau in other tauopathies

Only a handful of studies assessed the role of caspase-cleaved tau in non-AD tauopathies, and most studies had focused on tau Asp421 (D421). In PSP, appotosin, a mitochondrial carrier protein, activates caspase-3 and mediates tau cleavage at Asp421 [65]. Ferrer et al. showed TauC3 truncation in PSP in neurons but not in glia [66]. Guillozet-Bongaarts et al. also detected tau Asp421 in neurons but not in glia in AD and PiD [67]. On the other hand, Newman et al. showed tau Asp421 in PiD, PSP, and CBD within regions with neurofibrillary tangles, tufted astrocytes, and Pick bodies [68]. TauC3 was found in FTLD-tau/K317M tufted-like astrocytes and oligodendroglial inclusions [66]. In the same study using multiplex immunohistochemistry and novel tau-cleaved D402 and D13 mentioned above, Theofilas et al. showed that caspase-6 truncated tau is abundant in AD, to a lesser extent in PiD, and almost absent in 4R tauopathies (PSP, CBD, and AGD) in both neurons and glia [7]. Caspase-6 activation levels were also seen at much lower levels in 4R-tauopathies than in AD [7]. Taken together, these limited number of available studies show that although TauC3 is expected in the most common sporadic tauopathies, caspase-6 activation, and caspase-6 cleaved tau fragments are several levels of magnitude more predominant in 3R/4R and 3R tauopathies than in 4R tauopathies, making biomarkers based on caspase-6 cleaved tau potential tools to discriminate between AD and 4R-tauopathies.

## Caspase-cleaved tau fragments in CSF

Precise antemortem diagnosis of tauopathies poses a challenge, given the limited predictive accuracy of a neuropathological diagnosis based on the clinical syndrome for most neurodegenerative conditions. As an example, approximately one-third of cases that meet the clinical criteria for corticobasal syndrome exhibit AD pathology as their primary neuropathological feature finding. Furthermore, aside from AD, various conditions can underlie an amnestic syndrome, with limbic predominant age-related TDP-43 encephalopathy (LATE) being one of the most common in aging individuals [69]. The last decade saw an exponential increase in biomarker development aiming to enable differential diagnosis of tauopathies in vivo and monitoring tools to evaluate therapeutics. Most biomarkers for tauopathy are based on total tau and phospho-tau levels. The last decade saw an exponential increase in biomarker development aiming to enable differential diagnosis of tauopathies in vivo and monitoring tools to evaluate therapeutics. Most biomarkers for tauopathy are based on total tau and phospho-tau levels. Phospho-tau-based fluid biomarkers show good specificity and sensibility for detecting AD neuropathology, at least from moderate neuropathological stages [70, 71]. However, phospho-tau-based biomarkers proved to have limited utility in discriminating among tauopathies [72].

Studies on caspase-cleaved tau forms in cerebrospinal fluid (CSF) are limited, yet the emerging findings show promise, underscoring the need for deeper exploration. Using an enzyme-linked immunosorbent assay to detect caspase-6-cleaved tau at Asp402 in postmortem CSF (using a polyclonal antibody), Ramcharitar et al. showed *postmortem* CSF levels mirror caspase-6-cleaved tau levels and active caspase-6 immunohistochemistry in the hippocampal sections of the same AD individuals and caspase-6-cleaved tau CSF levels correlate with AD severity and lower scores in neuropsychological tests [6]. However, CSF assays based on the tau C-terminal are not ideal because CSF lacks C-terminal tau peptides, making it challenging to detect tau fragments above residue 254 [73–77]. The advancement of immunoassays designed for the detection of N-terminal cleaved-tau fragments holds the potential to serve as a diagnostic tool for Alzheimer’s disease (AD) and distinguish it from other tauopathies [75, 78]. Findings that NT1 fragments (consisting of the N-terminal sequence 6-198) measured in CSF can discriminate between AD and non-AD populations better than full-length tau or tau measured via the middle region alone [5]. Given caspase-6 cleaved tau abundance in AD but scarcity in 4R tauopathies, it is worth testing if a CSF assay for caspase-6 cleaved tau performs better in discriminating AD from 4R tauopathies than phospho-tau based assays. However, the probable most relevant use of a CSF assay for caspase-6 cleaved tau is to detect non-phospho-tau pathology in AD as it seems that neurons with D13 tau are abundant in AD and only partially overlap with phospho-tau in the same neurons [7], making detection of caspase-6 cleaved tau relevance for diagnostic and therapeutic uses. The most likely pertinent application of CSF assay for caspase-6 cleaved tau is identifying non-phospho-tau pathology in AD. This is supported by evidence suggesting an abundance of neurons with D13 tau in AD, which only partially overlaps with phospho-tau in the same neurons [7]. Thus, detecting caspase-6 cleaved tau holds significance for both diagnostic and therapeutic purposes in this context. While a specific biomarker using monoclonal antibodies against caspase-cleaved tau at the N-terminal is currently unavailable, the presented evidence lends strong support to the prospects of its future development. Particularly appealing would be a biomarker centered on caspase-6-cleaved tau at Asp13, given its N-terminal location and the existing availability of a monoclonal antibody [7].

## Caspase activation and implications for AD therapeutics

What triggers caspase activation in AD is undefined. Oxidative damage and even accumulation of alpha-synuclein in synucleinopathies result in mitochondrial dysfunction, leading to the release of cytochrome-c and caspase-9 activation, which activate the downstream effector caspase-3 [68]. Also, hydrogen peroxide species induce activation of caspase-3 and − 6, cleaving tau at Asp421 [79]. These two pathways would increase caspase-3 levels and facilitate tau cleavage [80]. When the activity of both caspases was blocked, the amount of cleaved tau was reduced significantly. Amyloid-β can activate caspases and cleave tau contributing to tangle pathology [26]. A study proposed a potential mechanism for activating caspase-8 by amyloid-β peptides in the brain of individuals with AD. The activation occurs via cross-linking with death receptors like Fas.

However, a study using primary human neurons that overexpressed wild-type or mutant APP challenged this model by linking the neurodegeneration process to caspase-6 instead of amyloid-β [42]. This finding suggests that caspase-6 can be activated independently of amyloid-β and at an earlier stage in AD. Moreover, the inhibition of caspase-6 slows caspase-3 activity, indicating a potential interaction between these enzymes. Thus, caspase-6 could potentially participate in activating caspase-3 and promoting the production of truncated tau at Asp421 [79].

Even with the mounting evidence implicating tau toxicity in AD pathogenesis, data from the AlzForum Foundation (www.alzforum.org) suggest that tau-targeting strategies constitute only 10% of the ongoing clinical trials for AD. Among these strategies, efforts to modulate the impact of caspase-truncated tau are relatively limited. For instance, one approach aims to alleviate the toxicity of truncated tau by inhibiting protease activity or selectively weakening protease-substrate interactions [64]. An alternative and attractive method centers around inhibiting caspase activation to reduce tau truncation. Drugs that inhibit caspases, such as minocycline and VX-765, are currently undergoing clinical trials for AD [58, 81–83]. Minocycline decreases levels of caspase-cleaved tau by inhibiting caspase-3 activation [83]. VX765, a blood-brain barrier permeable and likely non-toxic Casp1 inhibitor, blocks the Nlrp1-caspase1-caspase6 pathway, attenuating cognitive deficits and microglial activation caused by caspase-6 [59, 81, 82]. Efforts to develop highly selective caspase-6 inhibitors as a therapeutic approach for treating AD are also underway. The challenge in targeting caspase functions arises from the remarkable conservation of their active sites and catalytic machinery. To overcome this limitation, Van Horn and colleagues targeted a non-catalytic cysteine residue (C264) unique to caspase-6 to produce the first generation of a potent and irreversible caspase-6 inhibitor which exhibits selectivity over other caspase family members and high proteome selectivity [84]. Subsequent second and third-generation inhibitors, built upon this initial molecule, are being developed to enhance their potency and bioavailability. This is the starting point for the development of potent and isoform-selective inhibitors for caspase-6 as potential therapeutics. Caspase inhibitors may have the potential to treat tauopathies with significant pathological forms of 3R tau, but their effectiveness in treating 4R tauopathies is uncertain. Inhibiting the enzymes responsible for tau cleavage, such as caspase-6, may promote a significant therapeutic index since caspase-6 knockout mice are more resistant to pro-inflammatory and excitotoxic stimuli, have neuronal damage-induced microglial activation reduced, besides favorable outcomes in memory and neurological hallmarks [85, 86]. Further studies on caspase inhibitors are strongly encouraged.

## Conclusion and future directions

The advances in caspase-cleaved tau biomarkers and therapy promise an auspicious future for tauopathies research and move the field toward better diagnoses and disease-modifying events. The potential use of biomarkers in precision medicine is exciting, and caspase-cleaved tau in CSF may add the missing piece to track AD pathology *in vivo.* CSF D13 caspase-6-cleaved tau is the appealing biofluid biomarker to differentiate AD from 4R-tauopathies.

Our review reveals gaps in knowledge and overlooks significant aspects of the pathology of AD. Further research is needed to investigate the role of fragments produced by caspase-6 cleaved tau at D13 in AD. Additionally, it is crucial to understand in which stage caspase-6 cleaved tau is involved in the progression of AD, as well as the timing of the co-occurrence and dissociation between caspase-6 cleaved tau and phospho-tau pathology. Finally, in addition to developing specific inhibitor drugs for caspase-6, existing drugs could be repurposed to inhibit caspase-6 cleaved tau in AD and other tauopathies.

## Data Availability

Not applicable.

## Abbreviations

AD: Alzheimer’s Disease
AGD: Argyrophilic Grain Disease
APP: Amyloid Precursor Protein
ARTAG: Aging-related Tau Astrogliopathy
Asp: Aspartic Acid
CBD: Corticobasal Degeneration
CTE: Chronic Traumatic Encephalopathy
CSF: Cerebrospinal Fluid
GGT: Globular Glial Tauopathy
IL-1β: Interleukin-1 Beta
MCI: Mild Cognitive Impairment
MAPT: Microtubule-Associated Protein Tau
Nlrp1: NLR Family Pyrin Domain Containing 1
NLR: Nucleotide-Binding Oligomerization Domain, Leucine-Rich Repeat
NT1: N-Terminal 1
PiD: Pick’s Disease
PSP: Progressive Supranuclear Palsy
PTM: Post-Translational Modification
SAMP8: Senescence-Accelerated Mouse Prone 8
3R: Three Repeat
4R: Four Repeat
3R/4R: Three and Four Repeats
