# Publisher Correction: Exploring the significance of caspase-cleaved tau in tauopathies and as a complementary pathology to phospho-tau in Alzheimer’s disease: implications for biomarker development and therapeutic targeting

**DOI:** 10.1186/s40478-024-01772-5

**Published:** 2024-04-12

**Authors:** Liara Rizzi, Lea T. Grinberg

**Affiliations:** 1 Memory and Aging Center, Department of Neurology, Sandler Neurosciences Center, University of California San Francisco, 675 Nelson Rising Lane, 94158 San Francisco, CA USA; 2 Department of Neurology, University of Campinas (UNICAMP), Campinas, SP Brazil; 3 Department of Pathology, LIM-22, University of São Paulo Medical School, São Paulo, SP Brazil; 4 Department of Pathology, University of California San Francisco, San Francisco, CA USA

**Publisher Correction to:* acta neuropathol commun* 12**, **36 (2024)**

10.1186/s40478-024-01744-9

Following the publication of the original article [1], it was noted that due to a typesetting error the figure legends were paired incorrectly. The figure legends for Figs. 1 and 2 were wrongly given as captions for Fig. 2, 1 respectively.

The publisher apologizes for the inconvenience caused.

**Fig. 1:**
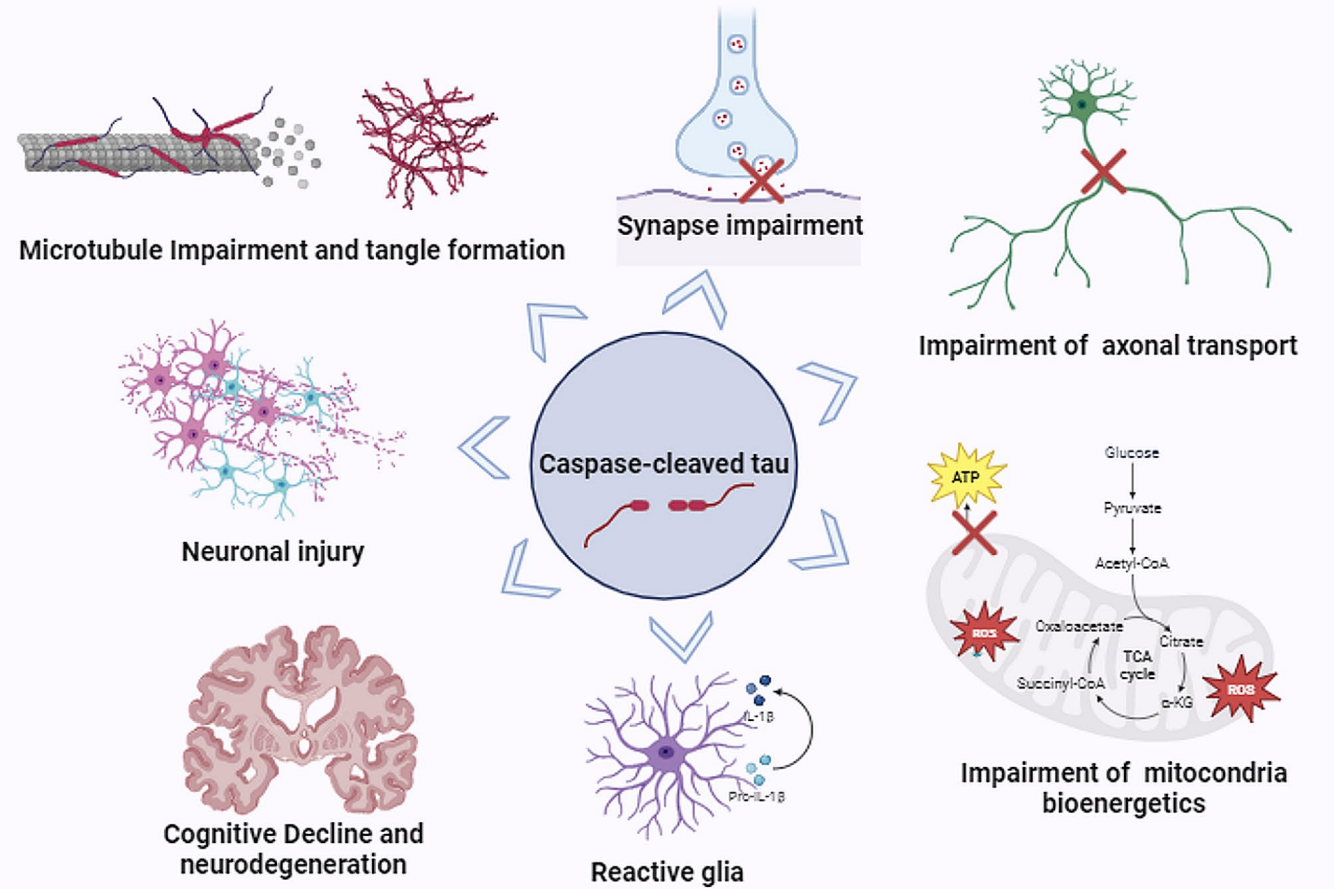
Pathological mechanisms induced by caspase-cleaved tau

**Fig. 2:**
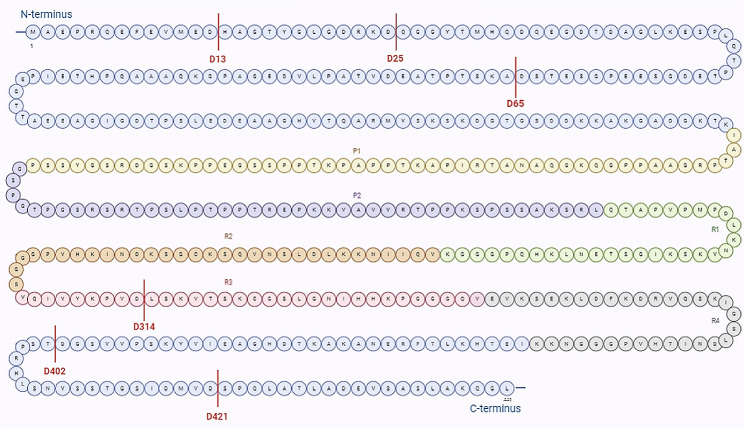
Putative sites caspase-cleaved tau. Caspases 1, 3, 6, 7, and 8 cleave tau at D421. Caspase-2 cleaves tau also at D65 and D314, caspase-3 cleaves tau also at D25, caspase-6 cleaves tau also at D402 and D13. Tau consists of four domains: the projection domain (M1–Y197), a proline-rich region (P1 and P2), the microtubule-binding repeats (R1, R2, R3, R4), and a C-terminus domain (K369–L441). Amino acids 1-441

The correct figures and captions have been included in this correction, and the original article [1] has been corrected.
